# Alternative polyadenylation: methods, mechanism, function, and role in cancer

**DOI:** 10.1186/s13046-021-01852-7

**Published:** 2021-02-01

**Authors:** Yi Zhang, Lian Liu, Qiongzi Qiu, Qing Zhou, Jinwang Ding, Yan Lu, Pengyuan Liu

**Affiliations:** 1 Department of Respiratory Medicine, Sir Run Run Shaw Hospital and Institute of Translational Medicine, Zhejiang University School of Medicine, Hangzhou, 310016 Zhejiang China; 2 Center for Uterine Cancer Diagnosis & Therapy Research of Zhejiang Province, Women’s Reproductive Health Key Laboratory of Zhejiang Province, Department of Gynecologic Oncology, Women’s Hospital and Institute of Translational Medicine, Zhejiang University School of Medicine, Hangzhou, 310006 Zhejiang China; 3 Department of Head and Neck Surgery, Cancer Hospital of the University of Chinese Academy of Sciences, Zhejiang Cancer Hospital, Key Laboratory of Head & Neck Cancer Translational Research of Zhejiang Province, Hangzhou, 310022 Zhejiang China; 4 Department of Physiology, Center of Systems Molecular Medicine, Medical College of Wisconsin, Milwaukee, WI 53226 USA; 5 Cancer Center, Zhejiang University, Hangzhou, 310029 Zhejiang China

**Keywords:** Alternative polyadenylation (APA), 3’UTR, Poly(A) sites usage, Cancer

## Abstract

Occurring in over 60% of human genes, alternative polyadenylation (APA) results in numerous transcripts with differing 3’ends, thus greatly expanding the diversity of mRNAs and of proteins derived from a single gene. As a key molecular mechanism, APA is involved in various gene regulation steps including mRNA maturation, mRNA stability, cellular RNA decay, and protein diversification. APA is frequently dysregulated in cancers leading to changes in oncogenes and tumor suppressor gene expressions. Recent studies have revealed various APA regulatory mechanisms that promote the development and progression of a number of human diseases, including cancer. Here, we provide an overview of four types of APA and their impacts on gene regulation. We focus particularly on the interaction of APA with microRNAs, RNA binding proteins and other related factors, the core pre-mRNA 3’end processing complex, and 3’UTR length change. We also describe next-generation sequencing methods and computational tools for use in poly(A) signal detection and APA repositories and databases. Finally, we summarize the current understanding of APA in cancer and provide our vision for future APA related research.

## Background

The maturation of nascent RNAs is a key step in transcription. For mRNA, the maturation of messenger RNA precursors (pre-mRNAs), involving the processing of 3’termini, is critical for mRNA function and stability [1]. In the processing of the 3’termini, the 3’end of nascent mRNA is cleaved, followed by addition of a poly(A) tail (i.e., polyadenylation). Polyadenylation protects the pre-mRNA from enzymatic degradation and facilitates nuclear export and translation [2]. The processing of poly(A) tail addition and length control of the poly(A) tail is modulated by polyadenylation polymerase and polyadenylation specificity factors [3]. Both cleavage and polyadenylation occur at polyadenylation sites (PASs) which are located within the 3’untranslated regions (3’UTRs), introns, or internal exons [4, 5]. Most eukaryotic genes contain multiple PASs. A conserved hexameric sequence AAUAAA [6], occurring upstream of the PASs, contains the most important signal (i.e., poly(A) signal) of pre-mRNA cleavage and polyadenylation. Both this canonical poly(A) signal and the PASs are widespread in eukaryotic mRNA. Cleavage or polyadenylation can generate transcript isoforms which differ in their coding regions or 3’UTRs [7]. This phenomenon, which gives rise to various transcript isoforms, is termed as alternative polyadenylation (APA).

Recent studies have shown that the global regulation of APA and the resulting distinct transcripts are involved in various aspects of tumorigenesis and cancer progression [8]. Differential PAS usage plays a key role in cell proliferation and gene versatility [9, 10]. For example, cell division cycle 6 (CDC6) is a critical gene in DNA replication. CDC6 can limit the rate of S-phase entry and regulate the initiation of DNA replication in mammalian cells [11]. CDC6 is upregulated in multiple human cancers and can inhibit the tumor suppressors p15^INK4b^, p16^INK4a^, and ARF [12]. Estrogen can induce the shortening of the 3’UTR of CDC6, and it has been observed that the resultant truncated isoforms can lead to aberrant expression of CDC6 via its avoidance of miRNA-mediated repression [13]. Such a 3’UTR length change does not simply occur in isolation on a certain gene but can be part of more global events in tumors or in certain other physiological conditions and contexts. Compared with normal cells, transcript isoforms in proliferated cancer cells are noted as having a tendency to be shortened [14], while transcript isoforms in senescent cells tend to be lengthened [15].

This review provides a general summary of four types of APA and their effects on gene regulation. We focus on APA regulatory mechanisms, including the interaction of APA with microRNAs, RNA binding proteins and other related factors, the core pre-mRNA 3’end processing complex, and 3’UTR length change. We also introduce high-throughput sequencing methods and computational tools for poly(A) signal detection and related corresponding additions to APA databases. Finally, we summarize recent research on APA in cancer and provide our vision for future APA related research.

## APA categories

**Fig. 1:**
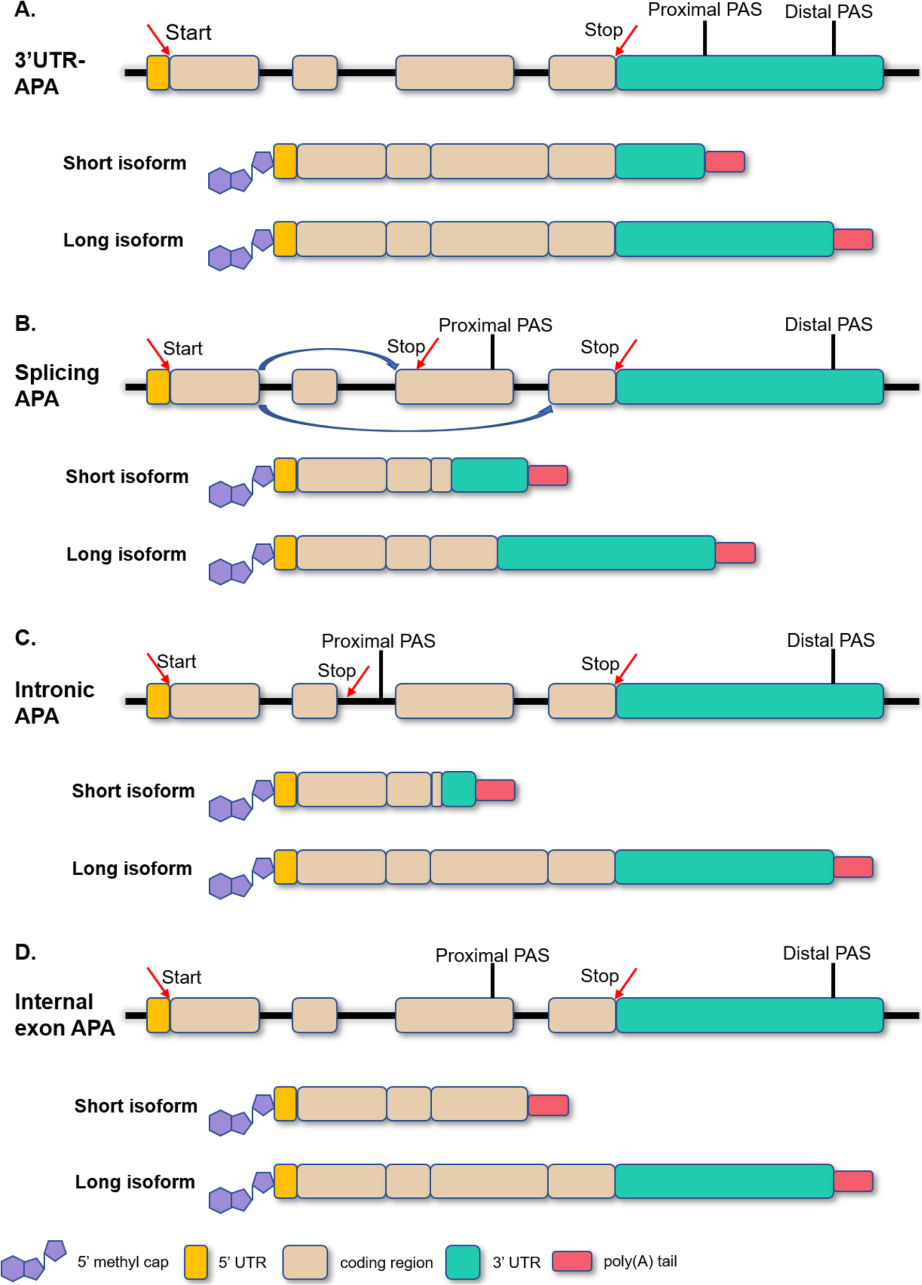
**Categories of APA. a** Tandem 3’UTR-APA containing two or more poly(A) sites in the 3’untranslated region. **b**, **c**, **d** UR-APAs occurring upstream of the last exon, therefore termed as an upstream region APA. **b** Splicing APA (alternative terminal exon APA) possessing a proximal PAS in the last exon and resulting in internal exon skipping. **c** Intronic APA occurring in the introns. **d** Internal exon APA generating a 3’UTR-lacking isoform via the PAS usage in the upstream exon

APA is a phenomenon that generates various transcript isoforms with different 3’termini from the same gene. It is observed in all eukaryotes species as an important mechanism of gene regulation. APA was first discovered in 1980 in the genes encoding immunoglobulin M (IgM) and dihydrofolate reductase (DHFR) [16, 17]. Over the next two decades, about 95 genes were identified as having APAs [18]. With the advent of next-generation sequencing (NGS) things accelerated greatly and by now more than two-thirds of human genes and one-third of mouse genes have been reported with more than one PAS containing a hexameric consensus motif AAUAAA, i.e., the canonical poly(A) signal [7, 19–22]. It is worth noting that the sequence AAUAAA (termed as poly(A) signal or pA signal) is different from the polyadenylation site (termed as poly(A) site or PAS). The poly(A) signal locates in upstream of the PAS. Undergoing diverse modifications, precursor RNAs with multiple PASs form into distinct isoforms. These can be divided into two subtypes according to the locations of the PASs (Fig. 1). One class of APAs are tandem 3’UTR-APAs, also known as 3’UTR-APAs, which contain two or more cleavage PASs in the 3’UTR and which generate various transcripts with different 3’UTR lengths. Tandem 3’UTR-APAs have a high number of incidences and have important impacts on mRNA stability, translation efficiency, nuclear export, cellular localization and localization of encoded protein. The other class of APA further changes the potential for protein-coding. This class occurs upstream of the last exon and thus is termed as upstream region APA (UR-APAs) [5, 23, 24]. It contains three subclasses, specifically, “alternative terminal exon APA” or “splicing APA” which generates transcripts with distinct 3’UTR sequences and encodes proteins with altered C-terminal amino acids; “Intronic APA” that occurs in an intron; and “Internal exon APA”, being the small fraction that appears in internal exons. These subtypes are involved in the cell-cycle and cell differentiation in many ways, such as in aspects of protein diversification and the inhibition of gene expression [25, 26].

### Tandem 3’UTR-APAs

Tandem 3’UTR-APA occurs in the 3’UTR and can change the structure of 3’UTRs or generate various isoforms of RNAs with different 3’UTR lengths (Fig. 1a). The longer the length of the 3’UTR, the more binding loci occur for microRNAs (miRNAs) and RNA-binding proteins (RBPs), and the more alternative RNA secondary structures are exhibited [4, 25, 27–29]. Like other cis-elements, these binding loci or RNA secondary structures can be specifically recognized by post-transcriptional factors and play important roles in gene regulation. Multiple mechanisms of gene regulation by 3’UTR-APA have been revealed. One major example is miRNA-mediated gene regulation at the 3’UTR of RNAs. Since 3’UTR-APA generates various 3’UTRs of different lengths, the number of miRNA binding sites in these isoforms is also different. The ability of miRNAs to down-regulate target genes varies with the number of binding sites, thereby affecting the stability and the translation of mRNAs [30].

Among these mechanisms, some are relevant to the progression and invasion of tumors. For example, GALNT5 uaRNA (a UTR-associated RNA) is a lncRNA derived from the 3’UTR of GALNT5. It promotes the proliferation of gastric cancer by interacting with the molecular chaperone HSP90 [31]. miRNA-200a reduces the level of PTEN expression by directly binding the 3’UTR of PTEN, thereby promoting the invasion of ovarian cancer cells [32]. These studies indicate that 3’UTR plays an important role in post-transcriptional gene regulation.

### UR-APA

UR-APA occurs upstream of the last exon, a location far removed from the 3’UTR. It can be further divided into three subclasses, namely alternative terminal exon APA, intronic APA, and internal exon APA. Alternative terminal exon APA occurs as a consequence of alternative splicing (Fig. 1b) [23, 24]. Both intronic and internal exon APA are components of mRNA decay pathways, including the non-stop decay pathway and nonsense-mediated decay pathway (Fig. 1c and d) [33–36]. Similar to 3’UTR-APA, UR-APA is also involved in many aspects of gene regulation.

## APA functions

### Interaction with miRNA

**Fig. 2:**
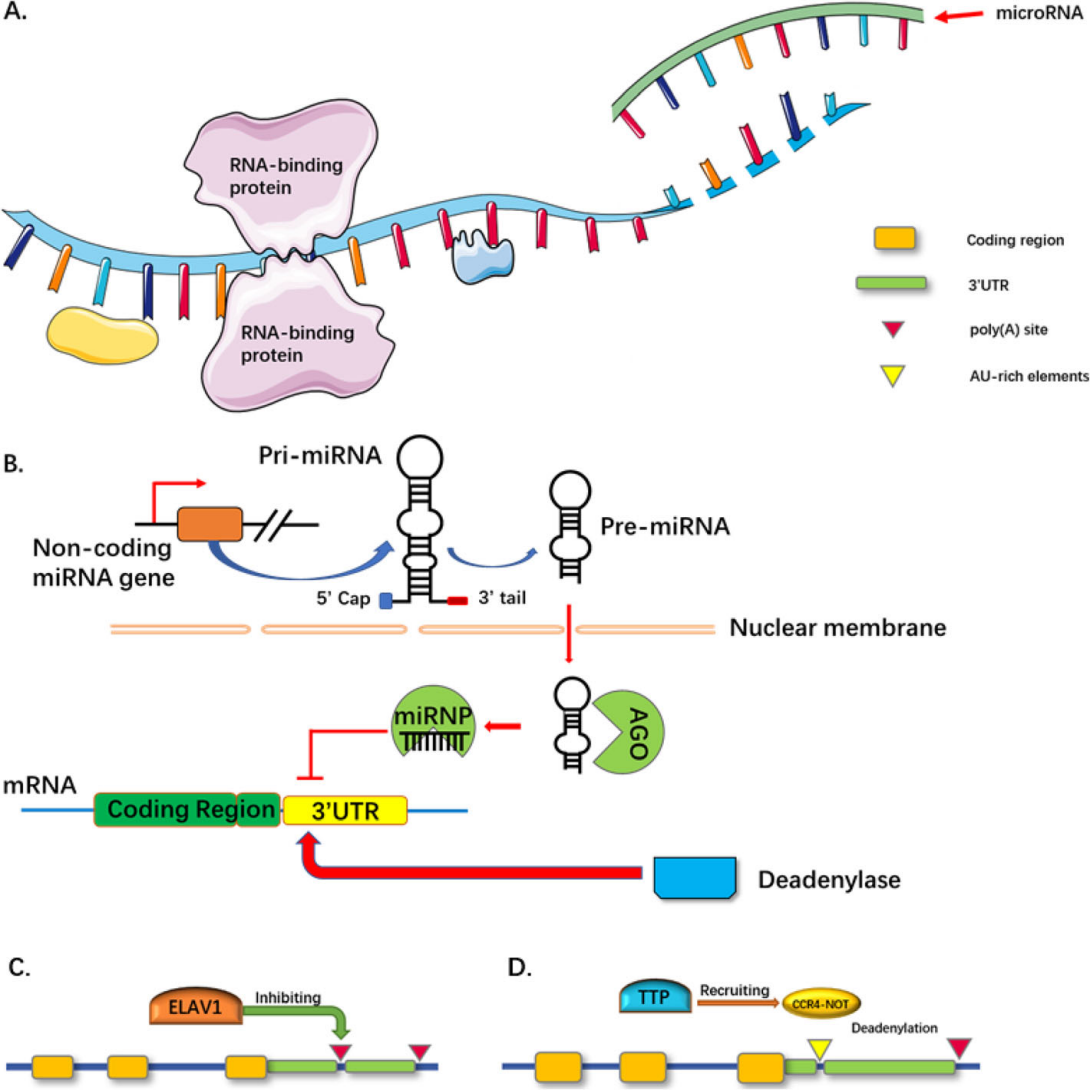
**APA functions.** A schematic diagram illustrating RNA-RBP interaction and RNA-miRNA interaction. **a** Multiple RBP binding sites and miRNA binding sites are located in the 3’UTR of RNA. As for the interaction between miRNA and 3’UTR, miRNA usually inhibits and silences the target RNA. **b** The scheme of RNA-miRNA interaction. MiRNAs can be firstly transcribed as long primary miRNA (pri-miRNA) transcripts with 5′ cap and 3’poly(A) tail by Pol II. Then pri-miRNA is cut by Drosha RNase III and turns into pre-miRNA in the nucleus. Pre-miRNA is delivered out the nuclei and processes into 21-nucleotide-long double-stranded RNAs. One strand combines with AGO proteins to form miRNA-containing RNPs (miRNPs). The miRNP complex binds to the complementary target mRNA and recruits deadenylase to repress translation. **c**, **d** RNA-RBP interactions. **c** ELAV leads to the expression of long 3’UTR isoforms during neurogenesis by inhibiting proximal PAS usage. **d** TTP recruits the CCR4-NOT complex into the ARE in the 3’UTR of the target gene and deadenylates the mRNA that causes its instability

miRNAs are a type of trans-acting element that can bind to the 3’UTR of mRNA and regulate gene expression at a post-transcriptional level [37–40]. They regulate the translation and stability of their binding mRNAs through translation inhibition and degradation of mRNA [41, 42]. Due to the existence of APAs in the 3’UTR, various isoforms with different 3′ termini are generated [43]. This mechanism can change which miRNA binding sites the 3’UTR contains (Fig. 2a and b). Distinct miRNAs targeting 3’UTR-APA were first discovered in cancer cells and activated T cells. Compared with the non-activated T cells and non-transformed cells, the length of 3’UTR in activated T cells and cancer cells becomes significantly shortened [44, 45]. Shorter 3’UTRs only possess proximal miRNA binding sites in male mouse germ cells, while those with longer 3’UTRs tend to contain distal miRNA binding sites [46]. Similarly, in-depth analysis of the 3’UTR isoforms of IGF2BP1 found nine functional PASs in human HLF cancer cell lines. Many of them have also been revealed to lack miRNA binding sites in these shortened isoforms [45]. This demonstrates that different numbers of miRNA binding sites occur among these 3’UTR isoforms and shows that differential PAS usage can be a clinical indicator for human disease. In addition, the reduction of miRNA binding sites is not the only consequence of 3’UTR shortening. Conserved miRNA binding sites are also seen to be preferentially enriched upstream of APA sites. 3’UTR shortening was found to be able to enhance the targeting efficiency of miRNAs that bind upstream of the proximal PAS [47]. Hence, 3’UTR shortening, resulting from APA, affects not only the number of miRNA binding sites within the 3’UTR, but also the targeting efficiency of miRNAs.

### Interaction with RNA-binding protein

The interaction between RNA and protein is essential for regulating gene expression at the post-transcriptional level (Fig. 2c and d). As a class of highly evolutionarily conserved proteins, RBP plays a key role in post-transcriptional gene regulation (PTGR) including aspects of maturation, stability, transport, and degradation of cellular RNAs. Most RBPs bind with mRNA and non-coding RNA, of which only ~ 2% are tissue-specific. RBPs are widely expressed and usually show higher expression levels than the average levels of cellular proteins [48–50]. The complex formed by RBP and RNA, ribonucleoprotein (RNP), is the major regulator in the PTGR. Defects in RBP function and RNP assembly are important causal factors leading to various human diseases including cancers. The types of RNA (e.g., mRNA, ribosomal RNA, and tRNA) that are predominantly bound by the RBPs lead to the characteristic phenotypes of these RBP related diseases [51–53].

RBPs contain specific RNA-binding domains (RBDs). These provide preferential selection of binding sites and targets and interact with RNA through these recognition regions. These RBDs include the RNA recognition motif (RRM), the K homology domain (KH), DEAD motif, double-stranded RNA-binding motif (DSRM), CCCH tandem zinc-finger domain, and Pumilio p-homology and Fem-3 mRNA binding factor (PUF) domains [48, 54–56]. Through their RRM, KH, and the zinc finger domains, the RBPs recognize Adenylate-undylate-rich elements (AREs), which are embedded in the 3’UTR and are present in 5–8% of human genes. These RBPs are called ARE-RBPs [57]. As in the miRNA binding sites, the altered number of the RBP binding motifs (such as AREs or GU-rich elements) caused by 3’UTR-APA can mediate mRNA stability. For example, the mRNA regulatory protein tristetraprolin (TTP, also known as ZFP36) can recruit the CCR4-NOT complex to the AREs in the 3’UTR of the target gene and then deadenylate mRNA, thereby destabilizing it. A lack of these AREs will result in an exceptional increase in mRNA expression [58–60]. As for TTP, the K homology splicing regulatory protein (KSRP) is another protein involved in mRNA degradation. Gherzi et al. showed that KSRP is an essential factor for ARE-directed mRNA decay. The depletion of KSRP results in the stabilization of several ARE-containing mRNAs such as TNFα and c-Fos. This stabilization is observed in KSRP-depleted S100 from several cell types, including Jurkat, HeLa, and HT1080 cells [61]. Furthermore, due to APA, human IFN-regulatory factor 5 (IRF5) has two isoforms with different 3’UTRs. The alternative expression levels of these two isoforms can cause systemic lupus erythematosus [62].

As can be seen from the above studies, the interaction between RBPs and the 3’UTR is deeply involved in PTGR and mRNA stability. It is often difficult to disassociate disease from transcription and translation. The regulation of RBP-RNA binding is a very important pathogenic mechanism of disease. For example, cold-inducible RNA binding protein (CIRP, also known as CIRBP or A18 hnRNP) is a stress-induced protein involved in cancer. CIRP can bind to the transcripts of pro-survival genes, which contain RNA signature motifs in their 3’UTRs, and stabilize them. In ectopic mouse xenograft models of human breast cancer and melanomas, CIRP promotes tumor growth by increasing the expression level of HIF-1α. Immunohistochemical analysis shows that CIRP is over expressed in the stroma and hypoxic areas of human tumors [63]. Furthermore, CIRP can also be transferred from the nucleus to the cytoplasm and bind to the 3’UTR of cyclin E1 mRNA and hTERT mRNA, thereby stabilizing and upregulating them [64]. Musashi (MSI) is another RNA binding protein, a mediator of a number of critical biological processes relevant to tumor initiation and progression. MSI was observed to be upregulated in many human cancer types, including colorectal, lung, and pancreatic cancers and glioblastomas. MSI regulates cancer invasion and metastasis through the regulation of mRNA stability and translation of proteins in several essential oncogenic signaling pathways, including those of NUMB/Notch, PTEN/mTOR, TGFβ/SMAD3, MYC, cMET, and others [65].

RBPs and RNAs assemble into a dynamic RNP complex. This plays an important role in RNA maturation, regulation, and transportation. Mutations in the heterogeneous nuclear RNPs (hnRNPs) cause amyotrophic lateral sclerosis (ALS) [66, 67]. Survival motor neuron 1 (SMN1) is one component of the small nuclear RNPs (snRNPs) assembly complex. Its loss of function directly affects the spliceosome and leads to spinal muscular atrophy [68]. The cyclin-dependent kinase inhibitor 1B (CDKN1B) mRNA is destabilized by the synergy of miR-221 and/or miR-222 and Pumilio homolog proteins (PUM) [69]. In *Drosophila melanogaster*, embryonic-lethal abnormal visual protein (ELAV) can be recruited to RNA polymerase II (Pol II) at promoter regions with GAGA sequences and then suspend Pol II. ELAV increases the expression of long 3’UTR isoforms during neurogenesis by inhibiting proximal PAS usage [70, 71]. All these studies indicate that not only that RBP expression, but also the type of RNA bound by the RBP, are involved in disease pathogenesis. These characteristic phenotypes and RBP factors could be investigated as potential novel markers for use in disease diagnosis and prognosis.

### Impacts on gene repression and versatility

UR-APA plays an important role in generating truncated transcripts. For example, Singh et al. showed that intronic APA isoforms, as widely expressed in immune cells and as participants in the development of B cells, lead to the production of truncated proteins lacking functional C-terminal domains. Furthermore, the number of intronic APA isoforms is decreased in multiple myeloma cells. This may contribute to the progression of multiple myelomas and is a factor associated with shorter progression-free survival [72]. A terminal exon characterization (TEC) tool has been developed for the analysis of RNA-sequencing data in order to identify isoforms ending at intronic poly(A) sites and to discover the prevalence of these APA isoforms [73]. A cleavage stimulation factor subunit named CSTF3 was seen with highly conserved intronic PASs which could lead to the production of severely truncated, probably nonfunctional, proteins [74]. This also involved a negative feedback regulation to reduce the expression of CSTF3 as a high expression level could induce the production of this UR-APA isoform. Similarly, retinoblastoma-binding protein 6 (RBBP6) has an isoform called Iso3, which is produced by the intronic APA of RBBP6. Iso3 is downregulated in several human cancers and can compete with normal RBBP6 for binding to core machinery, thereby inhibiting polyadenylation and regulating APA [75]. The truncated isoforms of Dicer and Forkhead box N3 (two tumor suppressor proteins), also lack tumor suppressive ability in tumors [76]. These studies suggest that truncated protein generation by UR-APA might represent a wide-spread gene inhibition mechanism.

On the other hand, the diversification of protein can also be a key part of gene versatility. For example, there are two isoforms of immunoglobulin M (IgM) heavy chain mRNA. The longer one, with the distal PAS usage in the 3’end of the third exon, is appropriate for membrane-binding, while the shorter one, with the proximal PAS in a composite terminal exon usage, is involved in secretion. Different mRNAs also predominate at different stages of immunocyte development, the longer ones at the lymphocyte stages and the shorter one at the secretion stages [10]. Another classic case is the calcitonin-related polypeptide-α gene (CALCA). CALCA has two transcript isoforms. The one with proximal PAS usage contains a skipped terminal exon and encodes the protein calcitonin. The other one, with distal PAS usage, generates an mRNA encoding calcitonin gene-related peptide 1 (CGRP). The expression of these two isoforms is tissue specific. Calcitonin mRNA is enriched in the thyroid and the other is enriched in the hypothalamus [77]. All these studies showed that UR-APA is a crucial ingredient of gene versatility and that, in many cases, each of these many isoforms of transcripts and proteins can perform unique functions.

## The core pre-mRNA 3’end processing complex

**Fig. 3:**
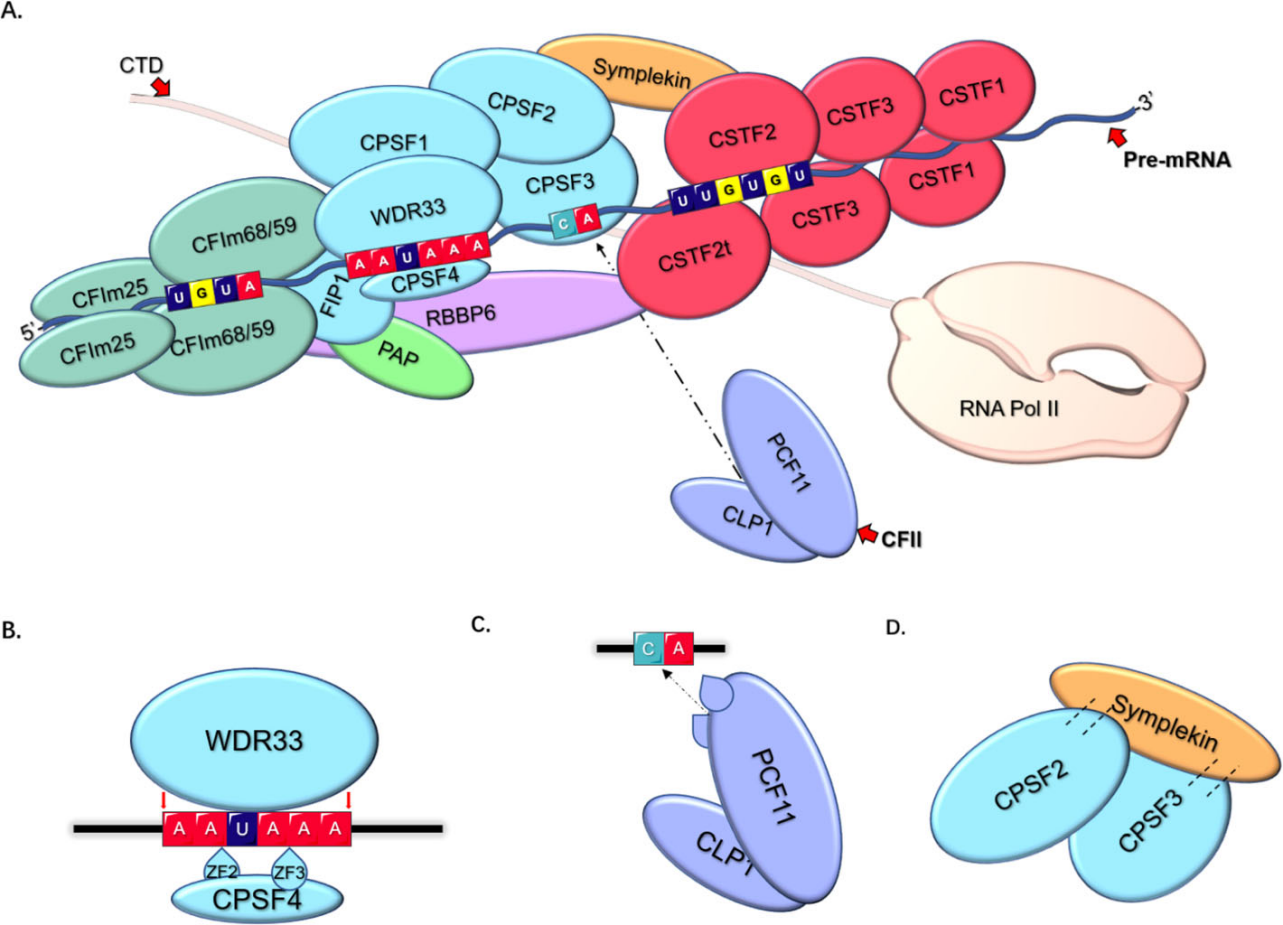
**Core pre-mRNA 3’end processing factors. a** The CPSF complex can recognize the AAUAAA hexamer and directly bind to the poly(A) site through CPSF4 and WDR33. CPSF3 is an endonuclease which preferentially targets cleavage sites containing CA elements. FIP1 binds to U-rich elements located upstream of the hexamer through its C-terminal domain, thereby modulating PAS recognition. It can also interact with PAP that is involved in cleavage. The CSTF complex is composed of dimers which can recognize and interact with U- and GU- rich elements downstream. CSTF can also interact with RBBP6, another important APA regulator. The CFI complex which contains CFIm68/59 and CFIm25, binds to the UGUA sequence as dimers in a similar manner to CSTF. As a part of the CFII complex it is responsible for the cleavage process. Both PAP and CFII are weakly or transiently involved in the pre-mRNA 3’end processing. Symplekin and RNA Pol II carboxy-terminal domain (CTD) have an impact on this interaction as scaffolds. **b** WDR33 recognizes the poly(A) signal and interacts with the AAUAAA hexamer directly. CPSF4 binds to the AAUAAA hexamer via its two zinc finger domains ZF2 and ZF3. **c** CLP1 and PCF11 interact via key residues of PCF11 which are highly conserved across eukaryotes. The mRNA binding is mediated by the two zinc finger domains of PCF11. The PCF11-CLP1 complex (CFII) targets the cleavage site which is located preferentially after a cytosine. **d** CPSF2, CPSF3 and symplekin can form a functional complex and interact with different accessory proteins to complete the maturation of pre-mRNAs

The core pre-mRNA 3’end processing complex contains four subcomplexes, namely cleavage and polyadenylation factor (CPSF), cleavage stimulation factor (CSTF), and cleavage factors I and II (CFI and CFII). These play a critical roles in APA formation and regulation (Fig. 3). Each of these will be introduced in detail in the following sections.

### CPSF

CPSF covers a class of regulators of PAS usage and a series of key proteins in pre-mRNA processing. The CPSF group contains CPSF1 (also known as CPSF160), CPSF2 (also known as CPSF100), CPSF3 (also known as CPSF73), CPSF4 (also known as CPSF30), FIP1 (also known as FIP1L1), and WDR33. It has been found that CPSF1 plays a key role in pre-mRNA 3’end formation. Recent studies have shown that the depletion of CPSF1 can induce cell cycle arrest at the G0/G1 phase and promote cell apoptosis in ovarian cancer cells [78]. Another study also indicated that the early-onset high myopia and retinal ganglion cell exon projection are related to CPSF1 [79]. In Arabidopsis, CPSF2 has been found to anchor poly(A) sites and mediate transcription termination [80]. CPSF2 can also be a prognostic marker for papillary thyroid carcinomas (PTC). In PTC patients, a lower expression of CPSF2 correlates with a worse prognosis [81]. As a pre-mRNA 3′-end-processing endonuclease, CPSF3 is involved in the termination of the transcript cycle, including RNA cleavage [82, 83]. CPSF4, a crucial subunit in this group, is closely related to tumor progression. For instance, CPSF4 can promote the growth and progression of lung cancer by targeting NF-κB/cyclooxygenase-2 signaling. In addition, CPSF4 is expressed aberrantly in colon cancer cells and then transcriptionally activates hTERT which facilitates colorectal tumorigenesis and development [84, 85]. FIP1 is a factor interacting with poly(A) polymerase (PAP). Via its C-terminal domain it can bind to the U-rich elements located upstream of the AAUAAA hexamer to modulate PAS recognition. FIP1 can also regulate APA in embryonic stem cells (ESCs) which is very important for ESC self-renewal [86, 87]. WDR33 is one of the main subunits of the AAUAAA hexamer binding factors in the mRNA 3’end processing in mammals, the other hexamer binding factor being CPSF4 [88, 89].

### CSTF

CSTF contains three subunits, CSTF1 (also known as CSTF50), CSTF2 (also known as CSTF64), and CSTF3 (also known as CSTF77). The CSTF complex can enhance CPSF’s recognition of upstream PASs. Specifically, CSTF1 plays a key role in the regulation of 3’end processing signal recognition. Studies have also shown that CSTF1 is involved in chromatin remodeling during DNA damage responses [90, 91]. CSTF2 has a paralogue named CSTF2t (also known as CstF64τ). Both forms are important in the promotion of the usage of non-canonical poly(A) sites. Knockdown of CSTF2 or CSTF2t will induce significant APA changes [92]. CSTF2 directly interacts with RNA via its RNA recognition motif, while the function of CSTF2t partially overlaps with CSTF2 [93]. CSTF3 is another crucial component of nuclear localization and polyadenylation [94]. In most cases, these three subunits are involved in the processing of mRNA 3’ends. For instance, CSTF1 is recruited to the CSTF to mediate the ability of PAS recognition by interacting with CSTF3, thereby increasing the affinity of CSTF2 for target RNAs. The Hinge domain of CSTF2 is essential for CSTF3 interaction [94, 95].

### CFI and CFII

CFI and CFII (also known as CFIm, CFIIm) are two core components of cleavage machinery and regulators of APA in mammals. CFI contains two small subunits of CFIm25 and two alternative large subunits of CFIm68 and/or CFIm59 [96]. CFI is a crucial regulator of 3’UTR length. CFI preferentially interacts with distal poly(A) sites in terminal exons to enhance distal PAS usage. It has been found that the CFI complex can help CPSF to interact with PASs more stably [97]. Furthermore, the loss-of-function of CFI, especially CFIm25 and CFIm68, leads to a transcriptome-wide increase in proximal PAS usage in HEK293 cells [98, 99]. CFII is the least characterized component of the 3’end processing machinery. CFII contains only two subunits, namely polyadenylation factor CLP1 (also known as hClp1) and PCF11. CLP1 controls the cleavage ability of CFII, whilst PCF11 affects the binding affinity of CFII with RNAs [100, 101].

### Other related factors

Other related factors can regulate APA and participate in the processing of its precursors, including poly(A) polymerase (PAP) complex (composed of PAPα and PAPγ), retinoblastoma-binding protein 6 (RBBP6), and others. For example, PAP is responsible for the efficient cleavage of PAS sites via the recruitment of FIP1 and CPSF1. PAP can also bind to an RBP-RNA complex called U1 small nuclear ribonucleoprotein (U1 snRNP) and inhibit polyadenylation [86, 102]. As a binding protein of p53 and Rb, the N-terminal of RBBP6 can interact with the CSTF complex and regulate APA processing [103, 104]. Di Giammartino found that the absence of RBBP6 in mammalian cells could lead to extensive 3’UTR lengthening and preferential inhibition of the usage of PASs containing AU-rich elements within their 3’UTRs [75]. Furthermore, scaffold symplekin and RNA Pol II carboxy-terminal domain (CTD) are noted as involved in the recruitment of polyadenylation regulators and seen to play a crucial role in the interaction between these core factors.

## 3’UTR length change

### 3’UTR shortening

**Fig. 4:**
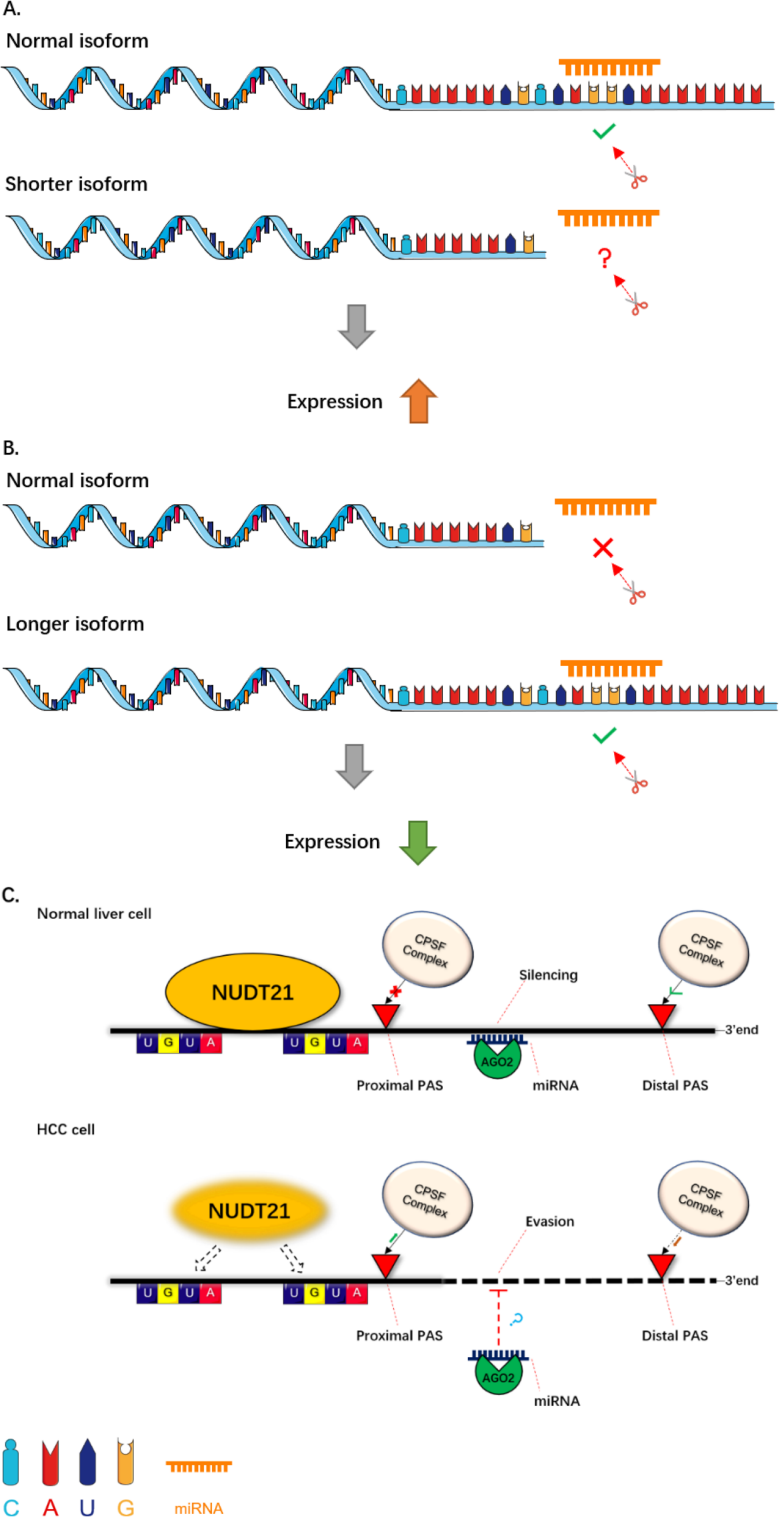
**3’UTR length change.** Dynamic mRNA isoforms with differential 3’UTR are generated by APA events. This is a schematic diagram illustrating two types of 3’UTR length change. **a** 3’UTR shortening. Various genes possess a tendency to generate shorter mRNA isoforms in tumors than in normal tissues. With the loss of miRNA target sites, the shorter isoform will escape miRNA-mediated decay, resulting in its aberrant up-regulation. **b** 3’UTR lengthening. In senescent cells, many genes possess a tendency to generate longer mRNA isoforms than in normal cells. With the use of distal PASs, the longer isoforms contain more miRNA binding sites and so are more likely to be silenced. This is a suppression mechanism to reduce the expression of genes. **c** An example of the APA regulation mechanism. In normal liver cells, an APA regulator NUDT21, which recognizes the 2 UGUA sequences upstream of the PAS, can protect the proximal poly(A) sites from cleavage of the CPSF complex. Therefore, the expression of the target gene can be regulated by AGO2-mediated miRNA. Conversely, the expression level of NUDT21 is downregulated in HCC cells. Lacking the protection of NUDT21, the proximal PAS is more likely to be recognized and cleaved by the CPSF complex than the distal PAS. Thus, the target gene can escape from the miRNA silencing due to lack of miRNA binding sites and thus express aberrantly [9]

3’UTR shortening is a significant consequence of APA regulation (Fig. 4a). On account of APA there are various transcripts with different 3’UTRs. The expression level of shorter transcripts can be increased via escaping miRNAs targeting their 3’UTRs [4]. In general, mRNAs with short 3’UTRs degrade more slowly than those of normal or lengthened subtypes. This may provide clues for identifying disease-related genes and uncovering key aspects of disease pathogenesis [105–107].

With the advent of NGS technologies, genome-wide profiling of APA sites has been performed in a variety of species, tissues, and disease states [105–107]. These studies have revealed that APA is a crucial regulatory mechanism for oncogene activation. Genes related to cell growth will be upregulated in proliferating cells by evading miRNA-mediated gene repression via their shortened 3’UTRs [25, 100]. Mayr and Bartel discovered a global enrichment of truncated transcript isoforms with shortened 3’UTRs in tumor tissues, in contrast to their adjacent normal tissues. These discoveries demonstrate that the truncation of mRNAs and the aberrant proteins caused by APA play crucial roles in tumor progression and invasion [30, 45]. Lembo et al. also found a strong correlation between 3’UTR shortening and the prognosis of breast cancer and lung cancer [30]. In a large sample analysis, Xia et al. identified 1346 genes from 358 pairs of tumor tissues and matched normal tissues in 7 tumor types of TCGA. The transcripts of these genes were generated by tumor-specific and recurrent APA. Most of these transcripts (~ 61–98%) displayed 3’UTR shortening in tumors [8]. In gastric cancer, Lai observed widespread 3’UTR shortening in more than 500 genes. Using a novel sequencing approach, this team identified ~ 28,000 poly(A) sites and revealed the potential connection between APA events and tumor metastasis. These shortened genes were mostly significantly enriched in the Rho GTPase pathway. The Rho GTPase pathway controls cytoskeletal regulation and represents important roles in the invasion of gastric cancer. Their study further demonstrated that NET1, a regulator of the Rho GTPase pathway, prefers proximal PAS usage in the MKN28 gastric cancer cell line with a high metastatic ability. Using a luciferase reporter assay, the shorter isoforms of NET1 were seen to exhibit a strong role in promoting transcriptional activity of the reporter gene in gastric cancer cell lines. Moreover, MKN28 cells transfected with short isoforms of NET1 had stronger capabilities of wound healing than those transfected with the longer isoforms [108]. These data provide strong evidence of the relevance of APA in cancer metastasis. Another recent study also found that 3’UTR-APA is enriched in triple-negative breast cancer (TNBC) and the shortening of 3’UTRs is more common in tumor tissues compared with normal breast tissues. This indicates that 3’UTR shortening can be a potential biomarker of TNBC recurrence and prognosis [109, 110]. Most of these genes with shortened 3’UTRs in tumor tissues are proliferation-related transcripts and are related to the clinical outcome of cancer patients, supporting the concept of APA-based proto-oncogene activation.

### 3’UTR lengthening

A wide-spread shortening of 3’UTRs in mRNAs by APA has recently been discovered in cancer cells. However, the post-transcription regulation of 3’UTR lengthening has not been fully illustrated (Fig. 4b). In 2018, Chen found global lengthening of 3’UTR in senescent cells due to APA. Genes that preferentially select distal PA sites in senescent cells are enriched in senescence-associated pathways [15]. The HNRNPA1-mediated 3’UTR lengthening of HN1 contributes to cancer- and senescence-associated phenotypes [111]. In a like manner, 3’UTR lengthening of Mdm2 can mediate the expression of p53, thereby contributing to cellular senescence in aged rat testis [112]. In addition to cellular senescence, 3’UTR lengthening also affects cell differentiation. 3’UTRs are reprogrammed by APAs during the generation of induced pluripotent stem (iPS) cells and the genes involved in this iPS cell generation were found to be more likely to exhibit 3′UTR lengthening [113]. As embryonic development progresses, mouse genes tend to express mRNAs with a longer 3’UTRs. This mechanistic regulation of 3’UTR-APA is coordinated with the onset of organogenesis and various aspects of embryonic development (including morphogenesis, differentiation, and proliferation) [114]. However, upstream factors controlling 3’UTR lengthening during cellular senescence and differentiation require further exploration.

### Global regulation of APA

Global 3’UTR regulation has been observed in various biological systems and processes including those of embryonic development, differentiation of myoblasts, and embryonic stem cells [114, 115]. For example, during the activation of primary murine CD4+ T lymphocytes a global decrease in the relative expression of distal 3′ UTRs was observed. This indicated that the 3’UTR was globally shortened [44]. This is consistent with the fact that transcripts with shorter 3’UTRs escape from miRNA targeting and thus increase their protein levels [116, 117]. Isoforms with proximal PAS usage that have greater translational potentials than others are generally upregulated when the membrane depolarization agents activate neurocytes [25, 118]. Another novel mechanism for global 3’UTR shortening is the activation of the mTOR pathway [119].

Global programs of APA-dependent isoform expression have been discovered in human cancers. Specific APA events have been implicated in various pathological conditions such as malignancies and autoimmune disorders. It has been hypothesized that the global regulation of polyadenylation activity might underlie the global APA profile changes. The usage of PASs is often altered in human hematological, immunological, and neurological diseases, as well as in cancers [8, 120]. There are various specific extracellular signals that can globally regulate APA. For instance, a poly(C)-binding protein named αCP was discovered as a global regulator of APA and a mediator of mRNA stability and translation [121, 122]. CSTF2 and CSTF2t are also essential global regulators of APA. CSTF2-RNA interactions are highly specific at PASs. Such interactions differ greatly in affinity and may be differentially required for PAS recognition. Furthermore, the co-depletion of the CSTF2 and CSTF2t can lead to striking APA changes, most of which are characterized by increased usages of distal PAS [123].

## Poly(A) signal detection

Since pre-mRNA isoforms with differing lengths of 3’UTRs are widely present in cells, many studies on the post-transcription regulation of pre-mRNA highlight the regulation of 3’UTR’s APA and poly(A) tail length changes. These studies not only reveal the mechanisms and factors that regulate cytoplasmic and nuclear changes in the poly(A) domain, but also clarify the relationship between these mechanisms. The relationship between 3’UTR-APA and miRNA targeting has been particularly illuminated. Similarly, relationships between deadenylation and the change of PAS usage in inflammation, or between cytoplasmic polyadenylation and the 3’UTR shortening in neurons, or relating to the alternative lengths of poly(A) tails in germ cells and tumors, have all been elucidated. As a novel mechanism for regulating various gene functions, APA has been involved in various biological processes including mammalian development, immune system function, disease pathogenesis, etc. [44, 124–128]. Hence, the detection of poly(A) signalling is very important for studying APA regulation and can be used as a powerful method to reveal disease pathogenesis and related aspects of diagnosis and treatment.

### Experimental methods for detecting APA

In 2014, two different high-throughput sequencing approaches were developed to sequence the 3′-terminome. Using the first of these methods, TAIL-Seq, researchers measured the length of the poly(A) tail and found the median poly(A) length to be 50-100 nt in HeLa and NIH 313 cells [129]. The second technique, Poly(A)-tail length profiling by sequencing (PAL-Seq), was first used to measure poly(A) tails of millions of individual RNAs in mouse livers, and zebrafish and frog embryos. It revealed an embryonic switch in translational control via APA regulation [130]. Soon after, an improved TAIL-Seq (mRNA-TAIL-seq, mTAIL-Seq) technique was developed, combining the strengths of TAIL-Seq and PAL-Seq. This was used to analyze poly(A) tails in *C. elegans*. The study revealed short poly(A) tails as a conserved feature of highly expressed genes [131]. Subsequent studies using these poly(A) sequencing methods revealed that the poly(A)-tail G-content and terminal uridylyltransferase regulate translational efficiency and the transcriptome [132, 133]. In 2015, another deep sequencing of mRNA 3′ termini (termed 3 T-Seq) was developed to identify APA events in gastric cancer cell lines. Using 3 T-Seq, researchers identified > 28,000 novel poly(A) sites, of which 513 genes had been observed to express shortened isoforms. They further characterized one of these 3′ UTR shortening genes, NET1, and found that the NET1 isoform with a short 3’UTR had stronger in vitro cell migration and invasion capabilities than that with a long 3’UTR, suggesting that APA plays a role in tumor metastasis [108]. More recently, two new APA detection methods based on single-cell RNA-seq, namely Full-length poly(A) and mRNA sequencing (FLAM-seq) [134] and Poly(A) inclusive RNA isoform sequencing (PAIso−seq) [135], have been developed. Using their new algorithm “tailfindr” [136], these new sequencing methods can detect poly(A) sites at a single-cell sensitivity and estimate poly(A) tail length from long-read sequencing data.

### Computational tools for detecting APA

In parallel with the advancement of experimental methods, computational tools to detect APA have been actively developed. These are summarized in Table 1. We will now introduce several of these popular tools that can complete the process from the sequence alignment to APA detection result.

**Table 1:** Computational tools for detecting APA

Name	Description	Environment	Year	Website	Ref.
InPAS	A package that can detect the dynamics of APA events from RNA-seq data by removing false sites due to internal-priming.	R	2013	http://www.bioconductor.org/packages/release/bioc/html/InPAS.html	[137]
ChangePoint	A change-point model based on a likelihood ratio test for detecting 3’UTR switching.	Java	2014	http://utr.sourceforge.net/	[13]
DaPars	A bioinformatics algorithm for the de novo identification of dynamic APAs from standard RNA-seq.	Python	2014	https://github.com/ZhengXia/dapars	[8]
Roar	A strategy for detecting alternative PAS usage and comparing these between two biological conditions.	R	2016	https://github.com/vodkatad/roar/	[138]
QAPA	An approach to infer and quantify APA from RNA-seq data.	Python & R	2018	https://www.github.com/morrislab/qapa	[139]
PAQR_KAPAC	A combined method that can quantify PAS usage from RNA-seq data and infer regulatory sequence motifs on PAS usage.	Python & R	2018	https://github.com/zavolanlab/PAQR_KAPAC.git	[120]
APAtrap	An approach to identify and quantify APA sites from RNA-seq data based on the mean squared error model.	R	2018	https://apatrap.sourceforge.io.	[140]
IntMap	An integrated method for detecting novel APA events from RNA-seq and PAS-seq data.	Matlab	2018	http://compbio.cs.umn.edu/IntMAP/	[141]
TAPAS	A tool that can detect more than two APA sites in a gene and APA sites before the last exon from RNA-seq data.	R	2018	https://github.com/arefeen/TAPAS	[142]
APARENT	A deep learning approach to predict APA from DNA sequences.	Python	2019	https://github.com/johli/aparent	[143]
DeepPASTA	A deep learning method to predict APA from DNA sequences and RNA secondary structure data.	Python	2019	https://github.com/arefeen/DeepPASTA	[144]
scDAPA	A tool to detect and visualize APA events from single-cell RNA-seq data.	R	2019	https://scdapa.sourceforge.io/	[145]
APAlyzer	A bioinformatics package which can examine 3’UTR-APA, intronic APA, and gene expression changes using RNA-seq data.	R	2020	https://bioconductor.org/packages/ release/bioc/html/APAlyzer.html	[146]
APA-Scan	A robust program that infers 3’UTR-APA events and visualizes the RNA-seq short-read coverage with gene annotations.	Python	2020	https://github.com/compbiolabucf/APA-Scan	[147]

DaPars is a powerful tool to identify numerous APA events from standard RNA-Seq data. It employs a piecewise linear regression to model read count data of RNA-seq to identify the location of the de novo proximal poly(A) sites. Using DaPars, Xia et al. identified 1346 genes with tumor-specific APAs from 358 pairs of tumor/normal samples across seven cancer types. Compared with normal tissue samples, more than 90% of these APA genes had shorter-length isoforms in the tumor samples. This tool has been widely used to detect APA events from RNA-seq data and has also been adopted by many databases [8].

APAtrap is capable of APA identification and quantification. Based on the mean squared error model, APAtrap can identify differential PAS usage and predict all potential poly(A) sites. When APAtrap was applied to the simulation data and real RNA-Seq data from human and Arabidopsis tissues, it showed higher accuracy than other tools in identifying APA events [140].

DeepPASTA is a deep neural network method to detect APA events. It was the first tool to predict poly(A) sites from both sequence and RNA secondary structure data. In addition, this tool can predict the most dominant poly(A) site of a gene in a specific tissue and predict the relative abundance of two polyA sites of the same gene [144].

Finally, scDAPA is a software package that can be used to detect APA profiles from single-cell RNA-seq data. It includes three main modules, namely 3’end annotation, APA event identification, and APA event visualization. scDAPA has a high degree of confidence for APA detection. This tool facilitates the portrait of dynamic APA profiles in different cell types from scRNA-seq data [145].

## APA databases

A large quantity of APA data has been produced using NGS techniques. Using these data, several databases have been established to facilitate the research community to obtain APA information from various samples. These are summarized in Table 2. In the following section, we introduce several major APA databases.

**Table 2:** APA databases

Name	Description	Year	Species	Website	Ref.
*PolyA-Seq Atlas*	A quantitative atlas of poly(A) sites using the PolyA-Seq protocol. Filtered sites are available via the UCSC Genome Browser.	2012	human, rhesus, dog, mouse, and rat	http://genome.ucsc.edu/	[19]
*APADB*	Database of APA sites and miRNA regulation events.	2014	human, chicken, and mouse	http://tools.genxpro.net/apadb/	[148]
*APASdb*	Database of APA sites and heterogeneous cleavage sites downstream of poly(A) signals.	2015	human, mouse and zebrafish	http://mosas.sysu.edu.cn/utr	[149]
*PolyA_DB3*	Database of cleavage and Poly(A) sites identified by the 3ʹREADS protocol.	2018	human, mouse, rat, and chicken	http://www.polya-db.org/v3	[150]
*TC3A*	Database of robust APA data from 10,537 tumors across 32 cancer types. It is focused on human cancers and utilizes routinely available large-scale RNA-Seq datasets from TCGA.	2018	human	http://tc3a.org	[151]
*PolyAsite2.0*	Web portal of poly(A) sites identified by all 3’end sequencing datasets.	2020	human, mouse and worm	https://polyasite.unibas.ch	[152]
*APAatlas*	Atlas of APA across a large number of normal human tissues from the Genotype-Tissue Expression project.	2020	human	https://hanlab.uth.edu/apa/	[153]

The PolyA_DB is a database for analyzing pre-mRNA cleavage and polyA sites. It contains a large amount of data on polyA sites in humans, mice, rats, and chickens. In 2018, this database had been updated to version 3.0 (renamed as PolyA_DB 3). Based on deep sequencing data, using the 3’READS method, this version contains large volumes of data from multiple samples to supplement PAS information. The database can also be visualized by the UCSC genome browser [150].

TC3A focuses on human cancers with large-scale RNA-Seq datasets from TCGA which contains 10,537 tumor samples across 32 cancer types and provides APA usage analysis and visualization. This atlas is based on a bioinformatics algorithm called DaPars and its updated version, DaPars2. Users can compare the PAS usage of genes between tumor and normal samples [151].

PolyASite is a resource of PAS information generated using 3’end sequencing in humans and mice. In 2019, it was updated to version 2.0 containing new PAS datasets from worm genomes. PolyASite 2.0 integrates sequencing data generated by multiple sequencing methods (such as 3’READS, SAPAS, PolyA-Seq, etc) [152].

The APAatlas contains 1,125,143 APA events from 9475 samples across a total of 53 human tissue types. It focuses on the APA events located in 3’UTR regions and provides a view of the APA landscape across tissues. APA events in the APAatlas were inferred using DaPars and SAAP-RS. Since the APAatlas includes a large amount of normal human tissue samples, compared with other databases, it contains more APA events from normal samples and provides a good opportunity for investigation of the correlation between PAS usage and gene expression [153].

## APA factors in cancer

Global APA within 3’UTR has been characterized in various cancer tissues and cells. Many of these are identified to be involved in the proliferation and metastasis of cancer cells. The following describes the role of several of these important APA factors in cancer (Table 3).

**Table 3:** APA factors in cancer

Factor	Subcellular location	Function	Biological Function	Related major cancer types	Ref.
***CIRP***	Nucleoplasm	Stabilizes transcripts of genes involved in cell survival and regulates the translational processing machinery.	Stress response	Renal cancer, endometrial cancer, lung cancer, pancreatic cancer, cervical cancer	[63, 64, 154]
***NUDT21***	Nuclear bodies and additionally in the centriolar satellite	Activates mRNA-processing by binding to 5′-UGUA-3′ elements located upstream of poly(A) signals and regulates gene expression in somatic cell fate through APA machinery.	Differentiation, mRNA processing	Liver cancer, bladder cancer, glioblastomas	[9, 154–156]
***PABPN1***	Nucleoplasm and additionally in nuclear speckles	Modulates the usage of poly(A) sites and controls the poly(A) tail length.	mRNA processing	Pancreatic cancer, liver cancer, renal cancer	[154, 157, 158]
***hnRNPC***	Nucleoplasm	Regulates the stability and translation level of mRNA.	mRNA processing, mRNA splicing	Ovarian cancer, breast cancer, lung cancer, liver cancer, renal cancer	[154, 159–165]
***RBBP6***	Nuclear speckles	Regulates DNA-replication and interacts with the p53/TP53-MDM2 complex as a scaffold.	DNA damage, DNA replication, Ubl conjugation pathway	Colorectal cancer, cervical carcinoma, myeloproliferative neoplasms	[75, 103, 154]
***CSTF2***	Nucleoplasm and additionally in nuclear bodies	Involved in the 3’end cleavage and polyadenylation of pre-mRNAs.	mRNA processing	Liver cancer, renal cancer	[92, 93, 154]
***PCF11***	Nucleoplasm and additionally in mitochondria	Involved in the degradation of the 3′ product of poly(A) site cleavage and Pol II transcription termination	mRNA processing	Urothelial cancer, head and neck cancer	[101, 154, 166, 167]
***U1 snRNP***	Nucleoplasm	Regulates the usage of poly(A) sites and controls the poly(A) tail length.	Ribonucleoprotein, RNA-binding	Pancreatic cancer, urothelial cancer, renal cancer	[102, 154, 168]

### NUDT21

Nudix Hydrolase 21 (also known as CFIm25 or CPSF5) encoded by the Nudt21 gene, belongs to the Nudix family of hydrolases [96]. This factor contains an RNA-binding functional region called the NUDIX hydrolase domain, which can help NUDT21 participate in PAS usage [169]. As a crucial regulator of APA, NUDT21 has been reported to be a tumor suppressor in human cancers. For example, in bladder cancer (BC), NUDT21 regulates the expression of ANXA2 and LIMK2 in the Wnt/β-catenin and NF-κB signaling pathways and inhibits tumor progression [155]. NUDT21 is downregulated in BC tumor tissues and its low expression is associated with poor prognosis for BC patients. NUDT21 overexpression inhibits cell growth, migration and invasion, whereas its knockdown exerts the opposite role in BC cells. Interestingly, a number of genes prefer distal PAS usage in NUDT21 overexpression cells, while they prefer proximal PAS usage in NUDT21 knockdown cells. ANXA2 and LIMK2 are two of these NUDT21-regulated genes through APA mechanism. In BC tumor tissues, downregulation of NUDT21 promotes the production of ANXA2 and LIMK2 transcripts with longer 3’UTRs, thereby reducing the expression of ANXA2 and LIMK2. The reduction in ANXA2 and LIMK2 expression inhibits the NF-κB and Wnt/β-catenin signaling pathways and thus promotes BC tumor progression [155]. Other studies have also found that NUDT21 is down-regulated in hepatocellular carcinomas (HCCs), where NUDT21 is involved in 3’UTR lengthening. Further, in normal liver cells, NUDT21 co-localizes with argonaute 2 (AGO2) in P/GW bodies. This interaction was diminished in HCCs leading to abnormal cell proliferation in HCC cases [9]. Another study also observed that the expression level of NUDT21 could affect the tumorigenicity of glioblastomas (GBMs) by regulating the 3’UTR-APA of Pak1 [156].

### PABPN1

Poly(A) binding protein nuclear 1 (PABPN1) plays a major role in the post-transcriptional processing of RNA and in controlling the poly(A) tail length of RNA transcripts. PABPN1 binds at proximal poly(A) sites to block their cleavage. Yu et al. characterized the APA profiles of 6398 patient samples across 17 cancer types from The Cancer Genome Atlas (TCGA) and of 739 cancer cell lines from the Cancer Cell Line Encyclopedia (CCLE). They identified 1971 clinically relevant APA events and their analysis further illustrated PABPN1 as a master modulator of 3’UTR shortening. PABPN1 possess the capacity of proximal PAS binding and then alters the APA site selection [170]. In triple-negative breast cancer (TNBC), Wang et al. identified 1631 significant APA events in 165 TNBC tissues and 33 matched adjacent normal tissues. Among these significant APA events, approximately 69% exhibited a preference for proximal PAS usage. This team identified CPSF1 and PABPN1 as two major regulators of APA events in TNBC using a pooled shRNA library screening. They then demonstrated that the tandem 3’UTR length of various genes is correlated with the expression level of CPSF1 and PABPN1. Knockdown of PABPN1 interferes with APA regulation, resulting in an extensive 3’UTR shortening in cell cycle related genes. Consequently, this inhibits cell proliferation and causes apoptosis and S phase arrest in TNBC cell lines [171]. In muscle cells, PABPN1 interacts with Matrin 3 (MATR3) and regulates RNA processing. Mutations in PABPN1 can also cause oculopharyngeal muscular dystrophy (OPMD) [157, 158].

### hnRNPC

Heterogeneous nuclear ribonucleoproteins C (hnRNPC) is an RNA-binding protein encoded by the HNRNPC gene in humans. hnRNPC regulates genome-wide PAS usage selection. By generating a pre-mRNA 3’end sequencing library from hnRNPC-knockdown cell lines, Gruber et al. observed that nearly 54% of PASs in exons had altered their usage from that of the control group. Mechanistically, hnRNPC binds the poly(U) motifs that are frequently located near distal poly(A) sites. HNRNPC’s binding in close proximity of distal poly(A) sites prevents them from cleavage and polyadenylation, thereby increasing genome-wide proximal PAS usage [172]. Aberrant up-regulation of hnRNPC has been observed in a variety of cancers or cancer cell lines including breast cancers, glioblastomas, hepatocellular carcinomas, ovarian cancers, and lung cancers [159–163, 173]. One recent study revealed that the up-regulation of hnRNPC plays a crucial role in establishing APA profiles that are characteristic for metastatic colon cancer cells. hnRNPC is responsible for the regulation of UTR-APA of a group of genes including MTHFD1L, which is closely related to cancer progression [164]. The level of hnRNPC expression is also related to clinical outcomes. Patients with a high levels of hnRNPC transcripts have poor overall survival and disease-free survival in human gastric cancers [165]. These studies suggest the potential of hnRNPC as a valuable prognostic biomarker and therapeutic target for cancer treatment.

### PCF11

As a part of CFII, PCF11 contains an N-terminal RNAPII C-terminal domain (CTD)-interacting domain (CID) and plays a role in transcription termination and mRNA nuclear export control [174, 175]. Li et al. showed that the depletion of PCF11 in mouse C2C12 cells led to global 3’UTR lengthening by APA [24]. PCF11, as a key APA regulator, has also been recognized as responsible for the extensive 3’end alterations observed in neuroblastomas. Postnatal down-regulation of PCF11 induces neurodifferentiation and a low expression of PCF11 is associated with a favorable outcome and spontaneous tumor regression in such neuroblastomas. Mechanistically, GNB1, a subunit of the Gβγ-complex, is an important modulator of Wnt signalling. It is mediated by PCF11 through APA regulation. In the presence of PCF11, the GNB1 transcript with short 3’UTR is predominant in neuroblastoma differentiation. The short isoform of GNB1 has higher translation efficiency and this corresponds to the higher expression level of the GNB1 protein, thereby leading to the suppression of Wnt signalling. The expression level of GNB1 becomes significantly reduced upon PCF11 depletion. All-trans retinoic acid (ATRA) is the first-line therapeutic drug for treating neuroblastomas. After neuroblastomas were treated with ATRA, the expression level of PCF11 was significantly reduced, confirming its anti-cancer effect [166]. These studies suggest that PCF11 is a major regulator of the APA process and an important modulator of Wnt signalling during the neuronal differentiation of neuroblastomas.

## Conclusions and perspective

Mounting evidence is now demonstrating APA as a new layer of regulation for gene expression. The four types of APA work synergistically with miRNAs, RBPs, and other factors, to regulate gene expression and functional versatility. Due to the differential usage of PASs, various transcript isoforms can be generated in cells. These transcript isoforms are involved in multiple cellular processes including control of the cell cycle, mRNA translation efficiency, and cell proliferation and differentiation. APA is frequently dysregulated in cancer and this promotes tumorigenesis and progression by increasing the expression of oncogenes and reducing the expression of tumor suppressor genes [45, 176–178]. It is worth noting that not all APA events have biological significance and the secondary poly(A) site can be important in development, differentiation and transformation processes. Some APA events may lead to cryptic unstable transcripts, many of which are rapidly degraded in cells [19]. Generally, the identification of biologically significant APA events involves computational prediction and statistical testing, followed by tailored in vitro and/or in vivo assays.

Many computational tools and databases have also been developed to detect APA events (Tables 1 and 2). Most of these infer information of PAS usage from standard RNA-seq data. Using deep learning models, some of these can predict novel APA events under different biological conditions. These tools make a great contribution to the analysis of genome-wide APA profiles, thereby greatly improving our understanding of the APA regulation of gene expression and functional versatility. However, these tools are mainly focused on tandem 3’UTR-APA. The potential impact of UR-APA, such as effects of internal exon APA on gene regulation, requires further exploration. It will be interesting to know whether 3’UTR-APA and UR-APA are mutually exclusive or co-occurr in genes, and to what extent they coordinate their respective regulation of genes to promote tumorigenesis and cancer progression. There is an urgent need to develop new computational tools tailored towards identifying UR-APA. Additionally, the direct sequencing of natural poly(A) RNAs by long-read sequencing technologies (such as Oxford Nanopore and Pacific Biosciences) [179–182] provides broad prospects for the further detection and quantification of these UR-APAs.

Extensive APA occurs during the pathophysiology of many diseases including cancers. In these, APA events are emerging as clinical biomarkers of high potential. Most of the differentially regulated APA events result in transcript isoforms with different lengths of 3’UTRs. These are often related to a variety of clinical characteristics. These APA events are independent of commonly used molecular data (e.g., gene expression and somatic mutations) [8], and have been found to associate with prognosis, recurrence, tumor subtypes, and staging in multiple cancer types [30, 45, 109, 110, 170]. Additionally, APA events are potential therapeutic targets for cancer treatment and clinical biomarkers for drug resistance. APA events are commonly observed in clinically actionable genes such as CTNNB1, PI3KR1, and FGFR2. PABPN1, an APA master regulator, regulates large numbers of clinically actionable genes. Associations between APA events and the sensitivities of FDA-approved anticancer drugs tested in cancer cells are also readily observable [170].

Although recent studies have greatly enriched our knowledge of APA, we still know little about certain functions such as the differential affinity of PASs, the recruitment of the 3’end processing complex and other details on the regulation of APA factors. Continuing in-depth research on the modulation of APA regulation, the impact of APA on biological processes, and the possibility of manipulating APA in disease treatment, remains of high priority.

## Data Availability

All data generated or analyzed during this study are included in this published article.

## Abbreviations

3’UTR: 3’Untranslated region
AGO2: Argonaute 2
ALS: Amyotrophic lateral sclerosis
ANXA2: Annexin A2
APA: Alternative polyadenylation
ARE: Adenylate-uridylate-rich element
BC: Bladder cancer
CALCA: Calcitonin-related polypeptide-α gene
CCLE: Cancer Cell Line Encyclopedia
CCR4-NOT: Carbon catabolite repression 4–negative on TATA-less
CDC6: Cell division cycle 6
CDKN1B: Cyclin-dependent kinase inhibitor 1B
CFI: Cleavage factors I
CFII: Cleavage factors II
c-Fos: Fos proto-oncogene
CGRP: Calcitonin gene-related peptide 1
CID: C-terminal domain (CTD)-interacting domain
CIRP: Cold-inducible RNA binding protein
CLP1: Cleavage factor polyribonucleotide kinase subunit 1
cMET: MET proto-oncogene
CPSF: Cleavage and polyadenylation factor
CSTF: Cleavage stimulation factor
CTD: Carboxy-terminal domain
CTNNB1: Catenin beta 1
DHFR: Dihydrofolate reductase
DSRM: Double-stranded RNA-binding motif
ELAV: Embryonic-lethal abnormal visual protein
ESC: Embryonic stem cell
FGFR2: Fibroblast growth factor receptor 2
FIP1: Cleavage polyadenylation factor subunit FIP1
GALNT5: Polypeptide N-acetylgalactosaminyltransferase 5
HCC: Hepatocellular carcinoma
HIF-1α: Hypoxia inducible factor 1 subunit alpha
HLF: Human Lung Fibroblasts
HN1: Hematological and neurological expressed 1
hnRNP: Heterogeneous nuclear RNP
HNRNPA1: Heterogeneous nuclear ribonucleoprotein A1
hnRNPC: Heterogeneous nuclear ribonucleoproteins C
hTERT: Telomerase reverse transcriptase in humans
IGF2BP1: Insulin like growth factor 2 mRNA binding protein 1
IgM: Immunoglobulin M
iPS cell: Induced pluripotent stem cell
IRF5: IFN-regulatory factor 5
KHdomain: K homology domain
KSRP: K homology splicing regulatory protein
LIMK2: LIM-domain kinase-2
MATR3: Matrin 3
Mdm2: MDM2 proto-oncogene
miRNA: MicroRNA
MSI: RNA-binding protein Musashi
MTHFD1L: Methylenetetrahydrofolate dehydrogenase (NADP+ dependent) 1 like
MYC: MYC proto-oncogene
NGS: Next-generation sequencing
NUDT21: Nudix Hydrolase 21
NUMB: NUMB endocytic adaptor protein
OPMD: Oculopharyngeal muscular dystrophy
PABPN1: Poly(A) binding protein nuclear 1
PAP: Poly(A) polymerase
PAS: Poly(A) site
pA signal: Poly(A) signal
PCF11: Cleavage and polyadenylation factor subunit PCF11
PIK3R1: Phosphoinositide-3-kinase regulatory subunit 1
Pol II: RNA polymerase II
pre-mRNA: Messenger RNA precursor
PTC: Papillary thyroid carcinoma
PTEN: Phosphatase and tensin homolog
PTGR: Post-transcriptional gene regulation
PUF: Pumilio p-homology and Fem-3 mRNA binding factor
PUM: Pumilio homologue proteins
RBBP6: Retinoblastoma-binding protein 6
RBD: RNA-binding domain
RBP: RNA-binding protein
RNP: Ribonucleoprotein
RRM: RNA recognition motif
SMAD: SMAD family
SMN1: Survival motor neuron 1
snRNP: Small nuclear RNP
TCGA: The Cancer Genome Atlas
TEC: Terminal exon characterization
TGFβ: Transforming growth factor beta
TNBC: Triple-negative breast cancer
TNFα: Tumor necrosis factor α
TTP: Tristetraprolin
U1 snRNP: U1 small nuclear ribonucleoprotein
uaRNA: UTR-associated RNA
UR-APA: Upstream region APA
WDR33: Cleavage polyadenylation factor subunit WDR33
